# Epstein-Barr virus LMP2A signaling *in statu nascendi *mimics a B cell antigen receptor-like activation signal

**DOI:** 10.1186/1478-811X-10-9

**Published:** 2012-04-03

**Authors:** Niklas Engels, Gökhan Yigit, Christoph H Emmerich, Dirk Czesnik, Detlev Schild, Jürgen Wienands

**Affiliations:** 1 Institute of Cellular and Molecular Immunology, Georg-August-University Göttingen, Humboldtallee 34, Göttingen 37073, Germany; 2 Institute of Neurophysiology and Cellular Biophysics, and DFG Center for Molecular Physiology of the Brain, Georg-August-University Göttingen, Humboldtallee 23, Göttingen 37073, Germany

**Keywords:** B Cells, Epstein-Barr virus, LMP2A, B cell antigen receptor, ITAM, tyrosine phosphorylation, Ca^2+^, latency, lytic replication

## Abstract

**Background:**

The latent membrane protein (LMP) 2A of Epstein-Barr virus (EBV) is expressed during different latency stages of EBV-infected B cells in which it triggers activation of cytoplasmic protein tyrosine kinases. Early studies revealed that an immunoreceptor tyrosine-based activation motif (ITAM) in the cytoplasmic N-terminus of LMP2A can trigger a transient increase of the cytosolic Ca^2+ ^concentration similar to that observed in antigen-activated B cells when expressed as a chimeric transmembrane receptor. Even so, LMP2A was subsequently ascribed an inhibitory rather than an activating function because its expression seemed to partially inhibit B cell antigen receptor (BCR) signaling in EBV-transformed B cell lines. However, the analysis of LMP2A signaling has been hampered by the lack of cellular model systems in which LMP2A can be studied without the influence of other EBV-encoded factors.

**Results:**

We have reanalyzed LMP2A signaling using B cells in which LMP2A is expressed in an inducible manner in the absence of any other EBV signaling protein. This allowed us for the first time to monitor LMP2A signaling *in statu nascendi *as it occurs during the EBV life cycle in vivo. We show that mere expression of LMP2A not only stimulated protein tyrosine kinases but also induced phospholipase C-γ2-mediated Ca^2+ ^oscillations followed by activation of the extracellular signal-regulated kinase (Erk) mitogen-activated protein kinase pathway and induction of the lytic EBV gene *bzlf1*. Furthermore, expression of the constitutively phosphorylated LMP2A ITAM modulated rather than inhibited BCR-induced Ca^2+ ^mobilization.

**Conclusion:**

Our data establish that LMP2A expression has a function beyond the putative inhibition of the BCR by generating a ligand-independent cellular activation signal that may provide a molecular switch for different EBV life cycle stages and most probably contributes to EBV-associated lymphoproliferative disorders.

## Background

A common feature of herpes viruses is their ability to maintain latent infections during which no virus particles are produced. The oncogenic Epstein-Barr virus (EBV) establishes such a latent infection in human B cells [1]. At least four different types of EBV latency have been described based on the expression patterns of EBV genes including those encoding latent membrane protein (LMP) 1 and 2A [2]. The lipid raft-resident LMP2A contains 12 transmembrane domains and both, the N- and C-terminus face the cytosol. An immunoreceptor tyrosine-based activation motif (ITAM) in the LMP2A N-terminus is constitutively phosphorylated and activates the protein tyrosine kinase (PTK) Syk [3]. This enables LMP2A to support development and maintenance of peripheral B cells in LMP2A transgenic mouse models [4,5]. We have previously shown that for these purposes LMP2A also employs the intracellular adapter protein SLP65 (BLNK or BASH), which is a key effector molecule of the B cell antigen receptor (BCR) [6]. Following engagement of the BCR, SLP65 in conjunction with the adaptor CIN85 nucleates assembly of the Ca^2+ ^initiation complex comprising Bruton's tyrosine kinase (Btk) and phospholipase C (PLC)-γ2 [7,8].

So far, the standard model system for biochemical analysis of LMP2A signaling mechanisms was based on EBV-transformed primary human B cells known as lymphoblastoid cell lines (LCL), which express, however, several EBV gene products. Although early studies demonstrated that the LMP2A ITAM in the context of chimeric transmembrane proteins activates the Ca^2+ ^initiation complex, experiments using LCL suggested that LMP2A acts as inhibitor of BCR-induced activation signals and prevents mobilization of Ca^2+ ^ions from intra- and extracellular sources [3,9,10]. This observation led to the hypothesis that LMP2A suppresses viral replication which would be induced upon BCR activation of LMP2A-negative cells [11]. However, recent studies showed that constant activation of BCR-regulated signaling pathways - as done by LMP2A - induces and maintains BCR unresponsiveness resulting in B cell anergy [12,13]. To circumvent this problem and to analyze LMP2A signaling *in statu nascendi *in non-anergic cells in the absence of any other EBV gene product, we now established a Cre/loxP-based system to inducibly express LMP2A in B cells. We show that expression of LMP2A not only activated PTKs but also the Ca^2+ ^initiation complex resulting in oscillatory Ca^2+ ^fluxes similar to those observed after BCR stimulation. This triggered activation of the mitogen-activated protein kinase (MAPK) pathway as well as the expression of EBV-encoded BZLF1, the master regulator of lytic EBV replication. In addition, the constitutively phosphorylated LMP2A ITAM modulated BCR-induced Ca^2+ ^mobilization. Our results show that similar to the antigen-activated BCR, induction of LMP2A expression can trigger the lytic EBV replication cycle in an ITAM-dependent manner. In contrast to the BCR, however, the LMP2A activation signal does not require extracellular ligation.

## Results

### Online monitoring of LMP2A signaling events using the cre/loxP recombination system

**Figure 1:**
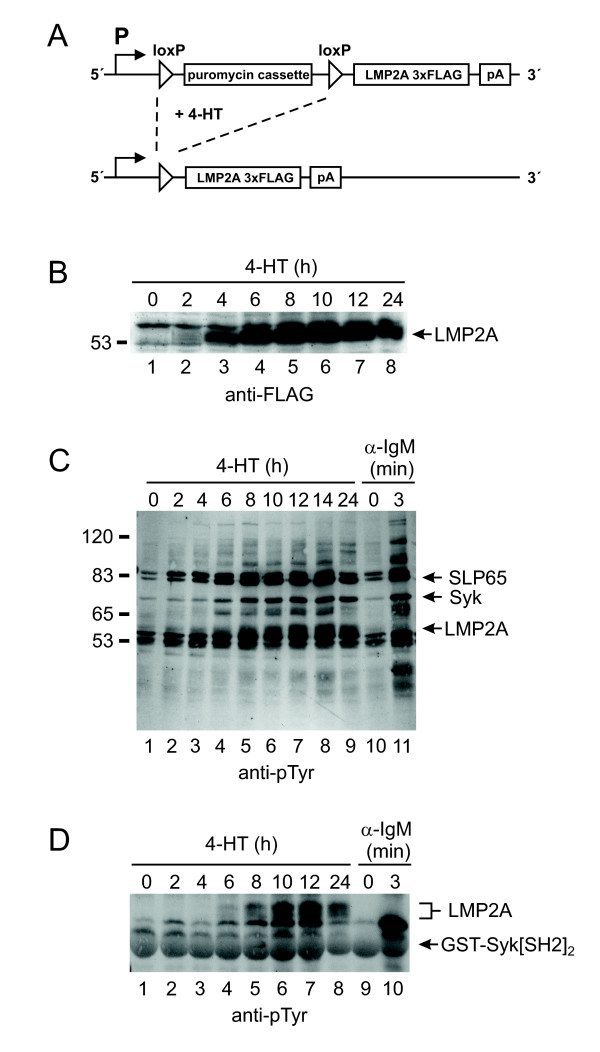
**Induced expression of LMP2A in DT40 B cells causes PTK substrate phosphorylation**. **(A) **LMP2A expression strategy. Treatment of DT40 transfectants with 4-HT induces Cre-mediated excision of a loxP-flanked puromycin resistance cassette which otherwise prevents the promotor (P) from activating transcription of a cDNA encoding triple-FLAG-tagged LMP2A (LMP2A3xF). **(B)**, Following 4-HT treatment for the indicated time points, expression of LMP2A3xF was monitored on the protein level by anti-FLAG immunoblotting of cellular lysates. **(C) **LMP2A transfectants of DT40 B cells were treated with 4-HT for the indicated times (lanes 1-9), left untreated (lane 10) or stimulated for 3 min with anti-IgM antibodies (lane 11). Upon lysis, tyrosine-phosphorylated proteins were purified using anti-phosphotyrosine antibodies (covalently coupled to NHS-Sepharose) and subjected to anti-phosphotyrosine (anti-pTyr) immunoblotting. Relative molecular masses of marker proteins are indicated on the left in kDa. (D) The same cells as before were treated with 4-HT for the indicated times (lanes 1-8), left untreated (lane 9) or stimulated for 3 min with anti-IgM antibodies (lane 10). After lysis in 1% NP40-containing lysis buffer, tyrosine-phosphorylated proteins were affinity purified using a fusion protein consisting of GST and the tandem SH2 domains of Syk (GST-Syk[SH2]_2_). The GST-Syk[SH2]_2 _fusion protein specifically binds to doubly phosphorylated ITAM motifs. Purified proteins were subjected to anti-phosphotyrosine immunoblotting. The positions of phosphorylated LMP2A and the (non-phosphorylated) GST fusion protein are indicated on the right.

In order to investigate the signaling capacity of LMP2A *in statu nascendi *during the first hours following its expression in the absence of other EBV-encoded molecules, we employed the Cre/loxP recombination system to express LMP2A in an inducible manner in DT40 B cells (for details see Methods and [14]). Briefly, treatment of the cells with 4-hydroxytamoxifen (4-HT) triggered Cre-mediated excision of a loxP site-flanked puromycin resistance cassette from the LMP2A expression vector (Figure 1A). This deletion brings the *lmp2a *cDNA under transcriptional control of a promotor. Anti-FLAG immunoblotting showed that LMP2A expression was detectable 4 h upon 4-HT treatment and increased thereafter (Figure 1B). A maximum was reached after an induction period of 12 to 24 h. Beyond this time, the amount of LMP2A declined continuously and was barely detectable after 48 h (data not shown). The exact molecular mechanisms that shut down LMP2A expression remain to be elucidated but this phenomenon could possibly explain why the generation of B cell transfectants that stably produce significant amounts of LMP2A is hardly feasible (data not shown). As shown in Figures 1C and 1D, induced expression of LMP2A activated PTKs and triggered phosphorylation of LMP2A itself as well as cellular substrates including the known downstream effectors Syk and SLP65 [3,6]. The increasing intensities of the anti-phosphotyrosine signals correlated with the amount of LMP2A protein, indicating that different LMP2A expression levels quantitatively and perhaps qualitatively regulate B cell signaling as suggested by Casola et al. [5]. Notably, the overall tyrosine phosphorylation pattern was very similar to that obtained upon BCR stimulation of LMP2A-negative control cells. Thus, mere expression of LMP2A is sufficient to constitutively activate B cell signaling cascades that are normally under BCR control.

**Figure 2:**
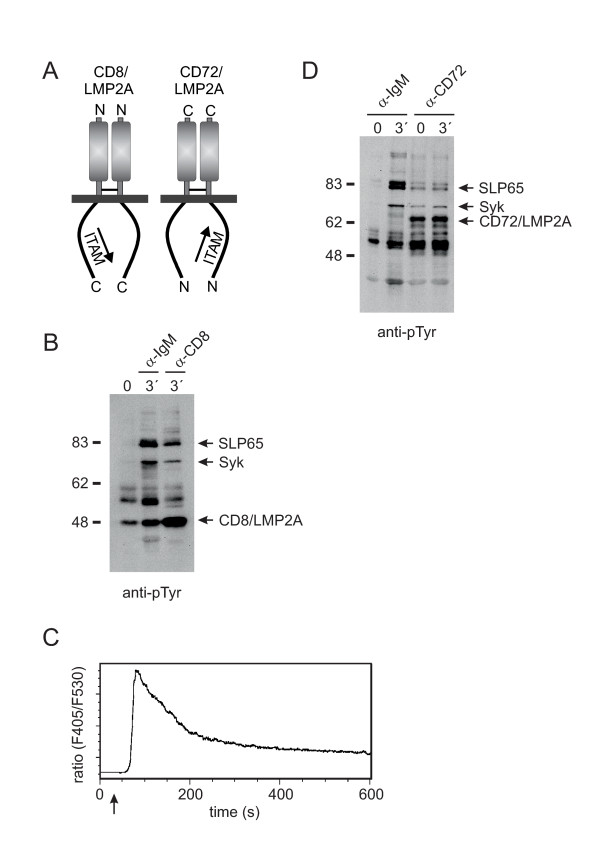
**Stimulation-dependent versus autonomous signaling of the LMP2A-ITAM**. **(A) **Schematic representation of CD8- and CD72-based transmembrane chimeras containing the cytoplasmic LMP2A N-terminus in type-I or type-II orientation, respectively. **(B) **DT40 transfectants, which constitutively express CD8/LMP2A were left unstimulated (lane 1) or stimulated for 3 min with either anti-IgM or anti-CD8 antibodies (lanes 2 and 3, respectively) and analyzed for PTK substrate phosphorylation by anti-phosphotyrosine immunoblotting as described in Figure 1C. Identification of Syk and SLP65 phosphoproteins was performed in separate experiments (data not shown). Relative molecular masses of marker proteins are indicated on the left in kDa. **(C) **Indo-1-loaded CD8/LMP2A transfectants were stimulated with anti-CD8 antibodies (arrow) and mobilization of Ca^2+ ^ions was monitored by flow cytometry. **(D) **DT40 transfectants, which were left uninduced (lanes 1-2) or treated with 4-HT for 6 h to induce expression of CD72/LMP2A (lanes 3-4) were analyzed for PTK substrate phosphorylation without stimulation (lanes 1 and 3) or after stimulation (lanes 2 and 4) with antibodies against IgM or CD72, respectively, by anti-phosphotyrosine immunoblotting as described in Figure 1C.

Early studies reported that when expressed in the context of chimeric transmembrane proteins the LMP2A ITAM required extracellular cross-linking to activate PTKs and subsequent Ca^2+ ^mobilization [15,16]. However, in those experiments the ITAM-containing N-terminus of LMP2A having a type-II like membrane topology was fused to type-I transmembrane proteins whereby its physiological orientation was inverted. To address a possible influence of the membrane topology on the need for extracellular ligation we analyzed the LMP2A N-terminus in the context of a type-I or a type-II transmembrane protein, respectively (see Figure 2A). Expression of a type-I CD8/LMP2A fusionprotein indeed required extracellular cross-linking to stimulate protein tyrosine phosphorylation (Figure 2B) and subsequent Ca^2+ ^mobilization (Figure 2C). In contrast - but similar to wild-type LMP2A - expression of the LMP2A N-terminus in the context of the type-II transmembrane protein CD72 caused the constitutive phosphorylation of intracellular signaling proteins which was not enhanced upon extracellular ligation (Figure 2D). In line with our experiences gained with wild-type LMP2A expression of the chimeric CD72/LMP2A fusionprotein could only be achieved using our conditional expression system (see Figure 1A and data not shown) which strengthens the notion that expression of a constitutively firing ITAM-containing receptor is not tolerated by DT40 B cells.

### LMP2A signaling mimics the activation signal of the BCR

**Figure 3:**
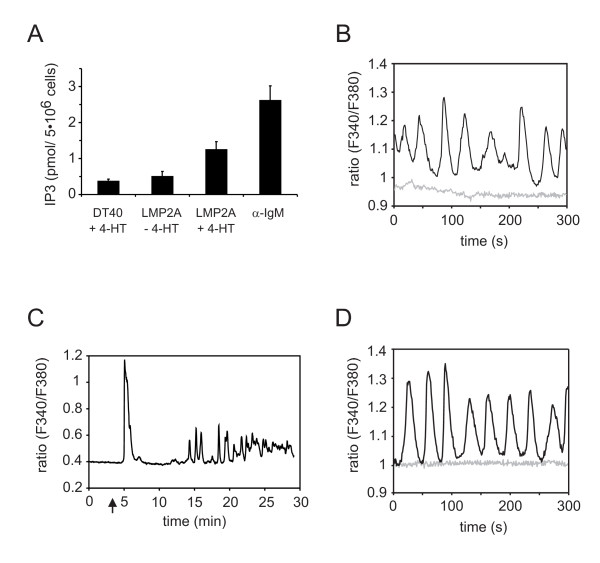
**LMP2A induces oscillatory Ca^2+ ^waves caused by IP3 production**. **(A) **DT40 cells that were left untreated (LMP2A -4-HT) or induced to express LMP2A for 12 h (LMP2A +4-HT) were subjected to IP3 analysis using a competitive binding assay with ^3^H-labeled IP3. Tamoxifen-treated parental cells (DT40 +4-HT) and cells that had been BCR-stimulated for three minutes (α-IgM) served as negative and positive control, respectively. Error bars represent standard deviation of four independent measurements. **(B) **DT40 B cells that were rendered positive for LMP2A expression by treatment with 4-HT for 14 h (black line) and LMP2A-negative DT40 cells treated with 4-HT for the same time (grey line) were loaded with Fura-2-AM. Subsequently, single cell [Ca^2+^] analysis giving the ratio of fluorescence intensities, F340/F380, was performed by microscopic imaging for 5 min. One representative record of 50 is shown for each sample. **(C) **LMP2A-negative DT40 cells were stimulated with anti-IgM antibodies (indicated by an arrow) and changes in intracellular Ca^2+ ^concentrations were monitored for 30 min at the single cell level. **(D) **Fura-2-AM-loaded DT40 cells that were induced to express chimeric LMP72 (black line) or 4-HT treated LMP72-negative parental cells (grey line) were monitored for Ca^2+ ^flux at the single cell level as described in **B**.

**Figure 4:**
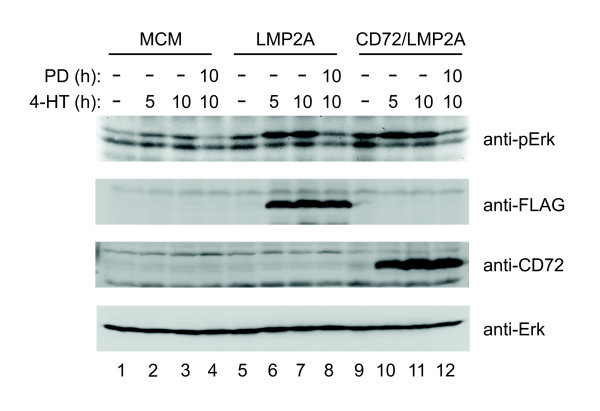
**LMP2A expression activates the Erk MAP kinase cascade**. DT40 cells were left untreated (lanes 1, 5 & 9) or induced to express LMP2A (lanes 6-8) or CD72/LMP2A (lanes 10-12) for the indicated times in the absence or presence of 50 M PD98059 (PD, lanes 4, 8 & 12). Parental cells served as control (MCM, lanes 1-4). Erk activation was monitored by immunoblot analysis of total cellular lysates with phospho-Erk-specific antibodies (upper panel). The membrane was sequentially re-probed with antibodies to the FLAG-tag, CD72 and Erk to demonstrate protein induction as well as equal protein loading.

Having established that mere expression of LMP2A or its cytosolic N-terminus in its physiological orientation activates cytosolic PTKs, we next examined whether the signal chain proceeded to the PLC-γ-controlled generation of second messengers. Measurement of inositol-1,4,5-trisphosphate (IP3) amounts in resting, LMP2A-expressing or BCR-activated cells showed that LMP2A signaling induced considerably increased steady-state amounts of IP3 that reached about half of what was present at the peak of the BCR response (Figure 3A). Single cell Ca^2+ ^flux analyses revealed that the elevated intracellular IP3 concentration gave rise to continuous cytosolic Ca^2+ ^spikes (Figure 3B) that mirrored those observed approximately 15 min after the initial peak in long-term measurements of BCR-activated cells (Figure 3C). Again, analysis of the CD72/LMP2A fusion protein paralleled our findings with wild-type LMP2A (Figure 3D). In addition, the Ca^2+ ^waves triggered by LMP2A were directly visualized by time lapse video imaging of Fluo-3-loaded cells (see Additional files 1, 2 and 3: Figure S1). The intensities of LMP2A-induced PTK activation and subsequent second messenger generation were sufficient to activate downstream signaling cascades such as the Erk MAP kinase pathway (Figure 4). Collectively, our data demonstrate that the LMP2A ITAM in its physiological membrane topology induces a ligation-independent activation signal during its initial expression that mimics that of the antigen-activated BCR.

### LMP2A signaling activates the EBV-encoded bzlf1 gene

**Figure 5:**
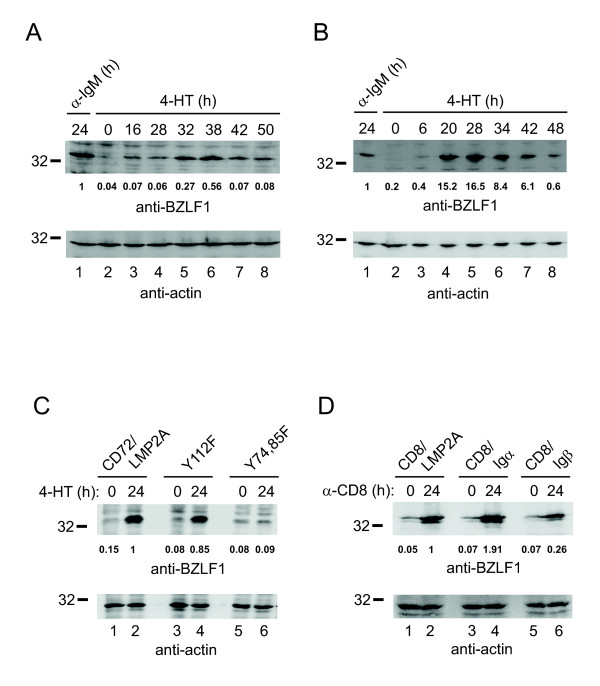
**LMP2A-mediated signaling induces expression of BZLF1**. DT40 transfectants stably transfected with a BZLF1 reporter construct (see text for details) were induced with 4-HT to express either wild-type LMP2A **(A) **or chimeric CD72/LMP2A **(B) **for the indicated times (lanes 2-8). As control, cells were cultured for 24 h in the presence of anti-IgM antibodies (lanes 1). Subsequently, cleared cellular lysates were subjected to immunoblot analysis with antibodies to BZLF1 or actin (upper and lower panels, respectively). **(C) **DT40 transfectants were left untreated or induced for 24 h to express either CD72/LMP2A (lanes 1- 2) or phenylalanine mutants Y112F (lanes 3-4) or Y74,85 F (lanes 5-6). BZLF1 protein expression was analyzed as described in **A**. **(D) **DT40 transfectants constitutively expressing chimeric CD8/LMP2A, CD8/Igα or CD8/Igβ, respectively, were either left untreated (0) or were stimulated with 10 μg/ml monoclonal anti-CD8 antibody for 24 h. Subsequently expression of BZLF1 and actin was analyzed as in **A**. Relative molecular mass of a marker protein is indicated on the left of the blots in kDa. Values below anti-BZLF1 blots indicate integrated optical densities of BZLF1 bands normalized to the corresponding actin bands.

To assess further downstream responses as well as the functional significance of LMP2A-induced signals we monitored transcriptional activation of EBV-encoded *bzlf1*, which is the physiological trigger of the lytic replication cycle in EBV-infected host cells [17]. A reporter gene assay was established in which the entire *bzlf1 *locus including pZ promotor, open reading frame and two potential polyadenylation sites was transfected into DT40 cells. With this experimental setup we recapitulated the in vivo situation of EBV-infected cells in which BCR-induced BZLF1 protein stimulates transcription of its own promotor in a feed-forward loop [18]. Indeed, following BCR activation of the DT40 transfectants for 24 h, BZLF1 protein production was detected by anti-BZLF1 immunoblotting (Figure 5A, lane 1). BZLF1 was also detectable following 16 h of 4-HT-mediated LMP2A induction and a maximum was reached after approximately 36 h (lanes 3-8). Note that, prior to feed-forward production of BZLF1, expression of LMP2A has to be induced for several hours by 4-HT treatment (see Figure 1) explaining the different kinetics of BCR- versus LMP2A-induced BZLF1 production. Likewise, the chimeric CD72/LMP2A protein, whose expression was induced with a faster kinetics than wild-type LMP2A (data not shown), triggered maximal BZLF1 production after induction for 20-28 h (Figure 5B). Mutational analysis of LMP2A signaling motifs showed that activation of the pZ promotor required a functional ITAM as exchange of its tyrosines to phenylalanine residues (Y74, 85 F) abrogated BZLF1 expression (Figure 5C, lanes 5-6). Inactivation of a non-ITAM tyrosine residue supposed to recruit the Src-family PTK Lyn (Y112F) only marginally affected LMP2A-induced production of BZLF1 (compare lanes 3-4 with 1-2). In contrast to the inducibly expressed LMP2A variants, the CD8/LMP2A fusion protein again required cross-linking with anti-CD8 antibodies to stimulate expression of BZLF1 in amounts similar to that achieved by the ITAMs of either Igα or Igβ (Figure 5D). Taken together, ligand independent LMP2A activation signals not only trigger cytosolic Ca^2+ ^waves and MAP kinase activation but in addition to this translate into nuclear responses at the crossroad of latency versus lytic EBV replication.

### LMP2A modulates BCR signaling

**Figure 6:**
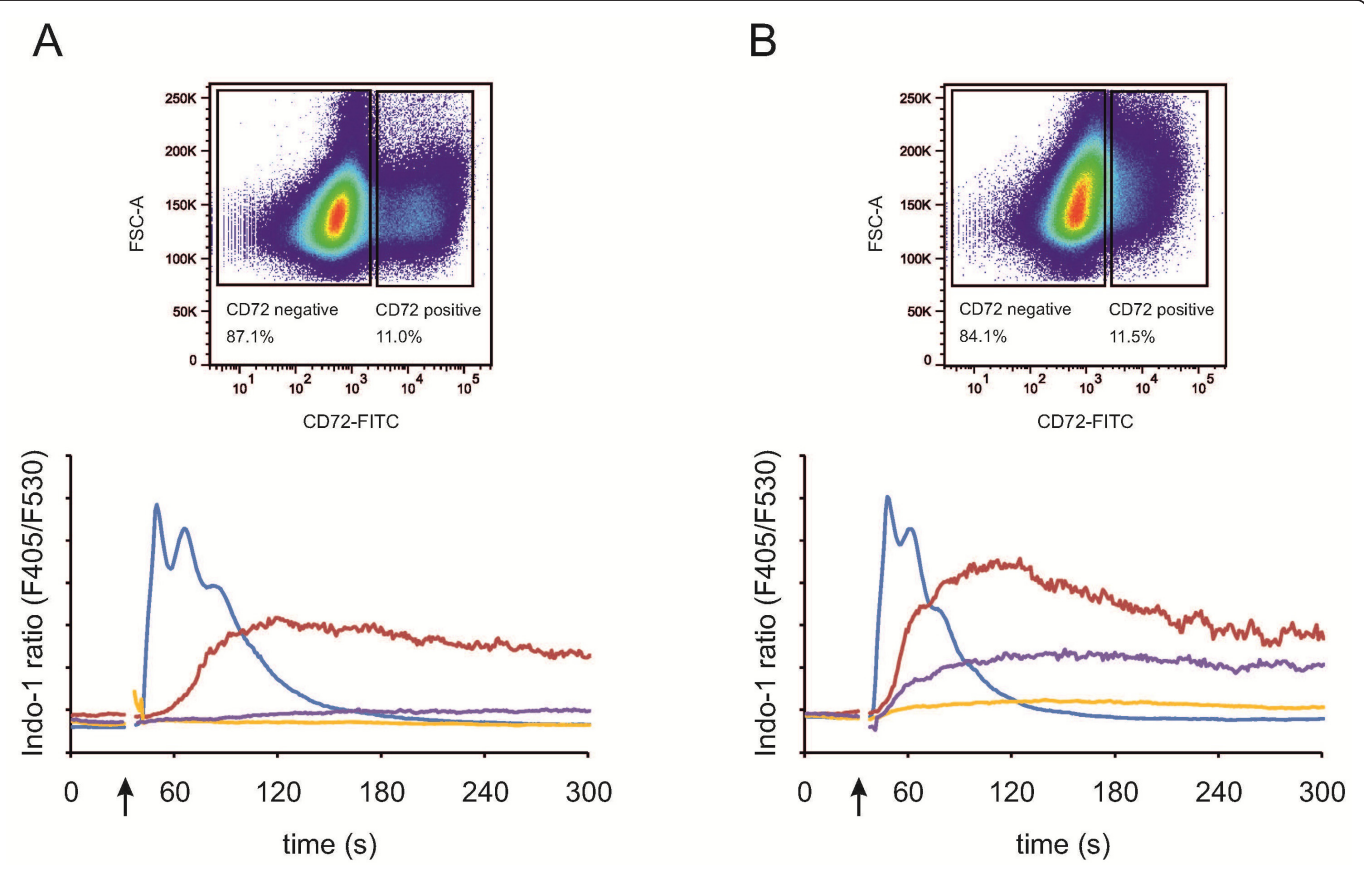
**The LMP2A N-terminus modulates BCR signaling**. DT40 transfectants were induced with 4-HT to express chimeric CD72/LMP2A, loaded with Indo-1 and stained with FITC-labeled anti-CD72 in order to gate on CD72 negative and CD72 positive cells (upper panels). Subsequently, BCR-induced Ca^2+ ^responses were monitored by flow cytometry (lower panels) upon stimulation of the cells with anti-IgM antibodies (blue and red curves) or with anti-mouse IgG F(ab)_2 _fragments to super-crosslink the anti-CD72 antibody (purple and yellow curves). Blue and yellow lines represent CD72/LMP2A-negative cells, red and purple lines CD72/LMP2A-positive cells. **(A) **and **(B) **represent two indepent cell clones.

Early observations made in LCL suggested that LMP2A may exert an inhibitory effect on BCR proximal signaling events. To test this possibility we compared BCR-induced Ca^2+ ^mobilization in B cells that did or did not express a constitutively active LMP2A ITAM (Figure 6). Since wild-type LMP2A was not detectable at the cell surface in our cells (data not shown) as reported for transiently transfected human B cells as well [19], we employed cells that were induced to express the CD72/LMP2A chimera. Expression of the chimera was detectable on the surface of live cells by staining with anti-CD72 antibodies (Figure 6A &6B). This allowed us to compare the BCR-induced Ca^2+ ^mobilization capacities of CD72/LMP2A-negative cells (blue lines) and CD72/LMP2A-positive cells (red lines).

Notably, the presence of the constitutively active LMP2A N-terminus drastically changed BCR-induced Ca^2+ ^kinetics. While it delayed and dampened the peak of the response it caused a sustained, long-lasting increase in the cytosolic Ca^2+ ^concentration compared with the relatively transient increase in wild-type cells. By contrast, crosslinking of CD72/LMP2A had only a minor effect on Ca^2+ ^mobilization (Figure 6, purple lines). These data demonstrate that expression of the constitutively tyrosine phosphorylated LMP2A N-terminus can influence BCR proximal signaling mechanisms if both molecules get active in the same cell at the same time.

## Discussion

With this report we reinforce the concept that LMP2A mimics BCR signal transduction [15,16]. The functional similarities are, however, not restricted to membrane-proximal signaling including PTK activation and phosphorylation of SLP65 as previoulsy thought, but extend to cytosolic Ca^2+ ^mobilization, activation of downstream signaling pathways and even nuclear responses such as activation of the EBV lytic gene *bzlf1*. These events are reminiscent of the antigen-induced BCR activation signal [7], which in fact represents a trigger of lytic EBV replication [20]. It thus appears that EBV possesses intrinsic and extrinsic modes for reactivation from latency, namely expression of and autonomous signaling by LMP2A as well as extracellular B cell stimulation through surface receptors such as the BCR.

The ligation-independent signaling capacity of LMP2A compelled us to employ an inducible expression strategy since permanent expression of the constitutively signaling LMP2A-ITAM was not endured by DT40 cells. The underlying molecular mechanisms that cause constitutive signaling of the LMP2A-ITAM remain elusive but are conserved in the type-II CD72/LMP2A fusion protein. This finding can explain the apparent discrepancy of studies made with chimeric type-I transmembrane proteins that required cross-linking with antibodies in order to signal and insights gained with full-length LMP2A in LCL.

The observed block of BCR signaling and *bzlf1 *transcription in LCL might be due to expression of additional EBV genes or a consequence of the cellular transformation process or both [21]. In LCL the latency type-III program is active, which normally operates only in proliferating EBV-positive B cells during infectious mononucleosis and post-transplantation lymphoproliferative disorders. This "EBV growth program" is characterized by the transcription of all EBV latency genes leading to the production of not only LMP2A but also LMP1, several Epstein-Barr nuclear antigens (EBNAs) as well as non-coding RNAs [22]. All of these molecules, perhaps in conjunction with the altered protein expression profile of transformed cells, may contribute to the particular LCL signaling phenotype. Furthermore, constant activation of BCR-regulated signal transducers leads to inhibitory feedback mechanisms that cause BCR unresponsiveness [13], a phenomenon that is most likely caused by continuous LMP2A expression alike. Nevertheless, our experiments show that the mere presence of the constitutively phosphorylated LMP2A N-terminus can modulate BCR proximal signaling events in non-anergic cells.

In the absence of a BCR, transgenic LMP2A expression can replace BCR signaling required for the development and survival of peripheral B cells [5,23,24]. As signaling for B cell survival requires a functional Ca^2+ ^machinery, i.e. SLP65 as well as enzymatically active Btk and PLC-γ2 [25-27], LMP2A must be able to trigger these molecules since otherwise it could not mediate development and maintenance of B cells in transgenic mice. As LMP2A signals autonomously without the need for extracellular ligation, it is most probably its amount produced in an individual cell that determines whether a maintenance signal or an activation signal is generated. Thus, moderate expression of LMP2A would drive B cell development and peripheral maintenance in transgenic mice as done by the unligated BCR. Elevated LMP2A expression by contrast mimics the activation signal of the antigen-stimulated BCR and leads to Ca^2+ ^mobilization and production of BZLF1. Indeed, when LMP2A was transiently transfected into non-anergic EBV-positive B cells, it induced entry into the EBV lytic cycle, which is associated with production of BZLF1 and Virus Capsid Antigen [28]. This may induce lytic EBV replication and contribute to the onset of severe lymphoproliferative disorders that are often associated with EBV infection especially in immunocompromised hosts [29,30].

## Conclusion

In conclusion, online monitoring of LMP2A expression and signaling in non-anergic B cells uncovered an activation mechanism for virus-intrinsic regulation of lytic genes involving second messenger-controlled cellular signaling pathways. The autonomously activated signal chains can activate *bzlf1 *transcription and thereby render the lytic EBV replication cycle independent of extrinisic regulators. Moreover, the activation signal emanating from LMP2A is likely to contribute to EBV-associated lymphoproliferative disorders like Hodgkin lymphoma and post transplantation lymphomas.

## Methods

### Plasmids and cDNAs

The *lmp2a *cDNA was amplified from an LCL (EBV strain B95-8). A triple FLAG-tag coding sequence was introduced by PCR into the coding sequence of the first extracellular loop resulting in LMP2A3xF which was ligated into pcDNA3 (Invitrogen) or pABESII. A puromycin resistance cassette flanked by two loxP sites was positioned between promotor and *lmp2a3xF *cDNA. Chimeric LMP2A/CD72 was generated by ligating the coding region of the LMP2A N-terminus (codons 1-121) to the coding sequence of transmembrane and extracellular parts of mouse CD72 (kindly provided by T. Tsubata, Tokyo, Japan). Chimeric CD8 constructs were generated alike (CD8 cDNA was kindly provided by B. Schraven, Magdeburg, Germany). The *bzlf1 *locus comprising promotor region (-221 onwards), open reading frame and two potential polyadenylation sites was PCR-amplified from an LCL (EBV strain B95-8) and ligated into pBS82HisD [31] carrying a histidinol resistance gene. Sequences of oligonucleotides are available upon request.

### Cells and antibodies

MerCreMer-expressing DT40 cells (kindly provided by T. Brummer and M. Reth, Freiburg, Germany) were maintained in RPMI1640 + GlutaMAX (Invitrogen) supplemented with 10% FCS and 1% chicken serum. Translocation of MerCreMer to the nucleus was induced by addition of 200 nM 4-hydroxytamoxifen (4-HT, Sigma, Munich, Germany). For stable transfections, 10^7 ^cells suspended in ice-cold PBS containing 30 μg of linearized expression vectors were electroporated using a Gene-Pulser (BioRad, Munich, Germany) (260 V, 960 μF in a 4 mm cuvette) and selected in the presence of 2 mg/ml G418, 1 mg/ml histidinol or 0.5 μg/ml puromycin, respectively. Monoclonal antibodies against BZLF1 (clone BZ.1) and human CD8 (MEM-31) were kind gifts of M. Rowe, Birmingham, UK or V. Horejsi, Prague, Czech Republic, respectively. Anti-FLAG (M2) was purchased from Sigma (Munich, Germany), anti-CD72 (K10.6) from BD Biosciences (Heidelberg, Germany), anti-phosphotyrosine (4G10) from Upstate (Biomol, Hamburg, Germany) and anti-phospho-Erk (E10) from CST (NEB, Frankfurt, Germany). Anti-chicken IgM antibody (M4) was purified from hybridoma supernatant. Prior to BCR stimulation, DT40 cells were starved for 30 min in RPMI without sera at 37°C and subsequently incubated with 3 μg/ml M4.

### Protein analysis

Cell lysis in 1% NP-40 buffer and immunopurification of cellular proteins have been described previously [6]. For specific immunodetection of FLAG-tagged LMP2A 10^6 ^cells were suspended in 30 μl Laemmli buffer, incubated for 2 min at 75°C and homogenized by repeatedly passing through a 26 gauge needle. Immunoblots were developed with ECL (GE Healthcare, Freiburg, Germany) using a digital image acquisition system (Intas Chemilux Entry, Intas, Göttingen, Germany). Densitometric analysis of individual protein bands on immuno blots was carried out with GelPro Analyzer software (Media Cybernetics, Silver Spring, MD, USA).

### Ratiometric Ca^2+ ^measurements and IP3 analysis

10^6 ^DT40 cells were loaded with either 1 μM Indo-1-AM or 0.25 μM Fura-2/AM (Molecular Probes) in RPMI containing 5% FCS and 0.015% pluronic acid at 30°C for 25 min. Subsequently, the cell suspension was diluted 2-fold with RPMI + 10% FCS and incubated for 10 min at 37°C. Cells were washed and resuspended in Krebs Ringer solution composed of 10 mM HEPES (pH 7.0), 140 mM NaCl, 4 mM KCl, 1 mM MgCl_2_, 1 mM CaCl_2 _and 10 mM glucose. After monitoring basal [Ca^2+^] levels for 1 min, cells were stimulated with 3 μg/ml M4. The ratio of Ca^2+ ^bound vs. Ca^2+ ^unbound Indo-1 fluorescence (405 nm/530 nm) was monitored on an LSR II cytometer (Becton Dickinson). Single cell ratiometric [Ca^2+^] measurements with Fura-2 were performed using an upright microscope (Axioskop 2, Zeiss, Göttingen, Germany) with a 40 × /0.9 water immersion objective. Alternating excitation wavelengths at 340 and 380 nm were provided by a custom-built monochromator consisting of a Xenon lamp, a galvanometer-driven mirror, appropriate filters, and a shutter. Image pairs F340/F380 (alternating excitation at 340 and 380 nm; emission > 505 nm) of individual cells were taken at 0.5 Hz or 1 Hz using a frame-transfer, back-illuminated CCD camera (16 bits/pixel, Micromax; Visitron, Munich, Germany) and a custom-built monochromator. Images were acquired at 100 ms exposure time per image. Galvanometer, shutter as well as the CCD's acquisition and recording software (Winview, Visitron) were synchronized by a custom-built microcontroller programmed in C language. Image analysis was performed using custom programs written in MatLab (The MathWorks, Natick, MA). IP3 amounts from 5 × 10^6 ^cells were determined using the D-*myo*-Inositol 1,4,5-trisphosphate [^3^H] Biotrak Assay System (GE Healthcare, Freiburg, Germany) according to the manufacturers instructions.

## Abbreviations

4-HT: 4-hydroxytamoxifen; BCR: B cell antigen receptor; Btk: Bruton's tyrosine kinase; BZLF1: BamHI Z Leftward reading Frame 1; CIN85: Cbl-interacting protein of 85 kDa; Cre: crossover recombination; DAG: diacylglycerol; EBNA: Epstein-Barr nuclear antigen; EBV: Epstein-Barr virus; Erk: Extracellular signal-regulated kinase; IP3: Inositol-1,4,5-trisphosphate; ITAM: Immunoreceptor tyrosine-based activation motif; LCL: Lymphoblastoid cell line; loxP: Locus of crossover in P1; LMP: Latent membrane protein; MAP: Mitogen-activated protein; MCM: MerCreMer; PLC-: Phospholipase C-; PTK: Protein tyrosine kinase; pTyr: Phospho-tyrosine; SLP65: SH2 domain-containing leukocyte protein of 65 kDa; Syk: Spleen tyrosine kinase

## Competing interests

The authors declare that they have no competing interests.

## Authors' contributions

NE designed the research, performed experiments and wrote the paper, GY and CE designed and performed experiments, DC and DS were involved in single cell Ca^2+ ^flux analysis, JW supervised the project and wrote the paper. All authors read and approved the final manuscript.

## Supplementary Material

Additional file 1 **Time Lapse Video Imaging of Ca^2+ ^Flux in LMP2A-expressing DT40 cells**. Click here for file

Additional file 2 **Figure S1A**. Click here for file

Additional file 3 **Figure S1B**. Click here for file
