# Supplementary material for: Epstein-Barr virus LMP2A signaling in statu nascendi mimics a B cell antigen receptor-like activation signal
Source: Cell Commun Signal. 2012 Apr 3;10:9. doi: 10.1186/1478-811X-10-9 (PMC3352256; doi:10.1186/1478-811X-10-9)
Supplement: Additional file 1 — Time Lapse Video Imaging of Ca2+ Flux in LMP2A-expressing DT40 cells. [file 1478-811X-10-9-S1.PDF]

Supplemental Data

Epstein-Barr virus LMP2A signalling *in statu nascendi* reveals a B cell antigen receptor-like activation signal

Niklas Engels, Gökhan Yigit, Christoph H. Emmerich, Dirk Czesnik, Detlev Schild and Jürgen Wienands

Supplemental Experimental Procedures

Cre/LoxP Recombination System

The Cre/LoxP recombination systems is based on DT40 B cells expressing a fusion protein encompassing a central Cre recombinase flanked on either side by a mutated version of the estrogen receptor hormone binding domain (MerCreMer), which is retained in the cytoplasm through association to HSP90. Upon treatment of the cells with 4-hydroxytamoxifen (4-HT) the MerCreMer fusionprotein detaches from HSP90 and enters the nucleus.

Confocal Microscopy and Time Lapse Video Imaging of Fluo3-loaded Cells

10^6 DT40 cells were loaded in 700 µl RPMI containing 5 % FCS, 1.5 µM Fluo3-AM (Molecular Probes) at 30°C for 25 min. Subsequently the cell suspension was diluted 2-fold with RPMI + 10 % FCS and incubated at 37°C for 10 min. Cells were washed twice and resuspended in Krebs Ringer solution containing 1 mM $CaCl_2$ and seeded onto Lab-Tek chamberslides (Nunc, Wiesbaden, Germany). Samples were examined on a Leica TCS SP confocal laser scanning microscope. Images were taken every 20 s for 30 min and converted into Quick Time Movies.

Supplemental Figures

Figure S1 (Quick Time Movies). Time Lapse Video Imaging of Ca^{2+} Flux in LMP2A-expressing DT40 cells

Cells were left untreated **(A)** or induced to express LMP2A with 4-HT for 14 hours **(B)**. Subsequently, cells were loaded with Fluo3-AM and Ca^{2+} mobilization was analyzed as described in Supplemental Experimental Procedures.
